# Maternal and neonatal factors associated with low birth weight among neonates delivered at the University of Gondar comprehensive specialized hospital, Northwest Ethiopia

**DOI:** 10.3389/fped.2022.899922

**Published:** 2022-08-15

**Authors:** Dagnew Getnet Adugna, Misganaw Gebrie Worku

**Affiliations:** Department of Human Anatomy, School of Medicine, College of Medicine and Health Science, University of Gondar, Gondar, Ethiopia

**Keywords:** low birth weight, prevalence, birth weight, newborns, factors, Ethiopia

## Abstract

**Introduction:**

Low birth weight is a major contributory factor to infant mortality. Although low birth weight remains an important public health problem in Ethiopia, little emphasis is paid to its intervention as a means of reducing neonatal mortality. The aim of this study was to assess the magnitude of low birth weight and its associated maternal and neonatal factors in newborns delivered at the University of Gondar Comprehensive Specialized Hospital.

**Methods:**

Hospital-based cross-sectional study was conducted, and 481 study participants were selected using systematic random sampling methods. Pre-tested interviewer-administered questionnaires were used to collect the data. Bivariable and multivariable binary logistic regression was implemented. Finally, the odds ratio with a 95% CI and a *p*-value of <0.05 were used to identify factors associated with low birth weight.

**Result:**

The prevalence of low birth weight was 12.5% (95% CI; 9.8, 15.7%). Preterm birth (AOR = 38; 95% CI: 15.3, 93.0), pregnancy-induced hypertension (PIH) (AOR = 2.6; 95%CI: 1.1, 6.4), maternal body mass index (BMI) of < 18.5 kg/m^2^ (AOR = 6.8; 95% CI: 1.5, 31.1), and grand multiparity (AOR = 4.2; 95% CI: 1.2, 16) were factors positively associated with low birth weight. However, babies delivered from mothers with age > 35 years (AOR = 0.14:95% CI 0.03, 0.7) had lower odds of low birth weight.

**Conclusion:**

In this study, the prevalence of low birth weight was higher than in the previous studies. The study revealed preterm birth, PIH, BMI of < 18.5 kg/m^2^, and grand multiparity were independent factors that increase the low birth weight while maternal age > 35 years reduces the low birth weight. Therefore, healthcare professionals should emphasize the early identification and management of women with PIH, tackling prematurity, and preventing maternal malnutrition through nutritional counseling as much as possible.

## Introduction

Birth weight is vital to the growth and developmental capacity of the infant, which is an important factor for child survival, disabilities, and stunting (1). Low birth weight often has long-term negative effects on the onset of chronic diseases in the course of life and thus needs effective public health measures (2). The WHO describes low birth weight as a birth weight of <2,500 g regardless of the gestational age, and it continues to be a major public health issue globally with several short and long-term adverse outcomes (3). Low birth weight incorporates both premature births (a birth of newborns before 37 weeks of gestation) and small for gestational age (birth weights less than 10*^th^* percentile for newborns of the same gestational age) (4–6). It can be caused by premature birth or the neonate being small for gestational age or both (5). It is a major underlying contributor to neonatal and infant mortality, which is responsible for almost half of all perinatal deaths (7). Globally, 15 to 20% of newborns have low birth weight, affecting more than 20 million births each year (1, 3, 8, 9). In Africa, the prevalence of low birth weight varies from 6.3 to 25.5% (10–13). In Ethiopia, the magnitude of low birth weight varied greatly ranging from 7.8 to 54% (14–18). The WHO has noted that most low birth weight neonates are delivered in developing countries; of these, 90% of them were in sub-Saharan Africa (19). Low birth weight remains the main public health concern due to poverty and other social factors such as lower socioeconomic level, lack of nutrition, and prenatal care (20). Factors that may raise the risk of developing low birth weight in addition to premature birth and intrauterine growth retardation (IUGR) include infection during pregnancy, inadequate weight gain, previous pregnancy with a low birth weight infant, smoking, alcohol or drug use, and age of the mother (21–23). In 2012, World Health Assembly’s global nutrition goal planned to decrease the prevalence of LBW by 30% between 2012 and 2025 (24). The first important step in designing effective management strategies is to recognize predictors of low birth weight. According to various studies, socio-demographic, obstetrics, and fetal factors are associated with low birth weight. This includes maternal age, multiple births, pregnancy-induced hypertension, obstetric complications, chronic medical disorders, and nutritional status (11, 25–30). Besides, the educational status of the partner, lack of antenatal care (ANC) visit, history of obstetric problems, maternal weight during pregnancy, short birth interval, and gravidity were factors significantly correlated with low birth weight (7, 8, 27, 31–37). This forecasts the future wellbeing, development, and viability of the child and is a strong overview of several public health concerns such as long-term maternal malnutrition and inadequate healthcare throughout pregnancy (31). Low birth weight is still an important public health concern in Ethiopia. However, in most developing countries, namely, Ethiopia, little emphasis is given to low birth weight intervention as a means of reducing neonatal mortality. Although there is some evidence of low birth weight in some parts of Ethiopia, there is a great discrepancy in the prevalence and factors that affect low birth weight in different geographical regions and periods. Also, most previous studies did not consider the methodological issue including appropriate sample size calculation and sampling technique. As a result, the study addresses this methodological gap which other studies did not consider (for instance, the previous study conducted in Gondar used a retrospective study with a small sample size of 240). Therefore, the aim of this study was to assess the magnitude of low birth weight and its associated maternal and neonatal factors at the University of Gondar comprehensive specialized hospital. The findings of this study may help policymakers, obstetric care providers, and program managers to design an intervention for preventing low birth weight.

## Materials and methods

This study was conducted at the University of Gondar Comprehensive Specialized Hospital, Gondar town, Northwest Ethiopia. It is one of the largest teaching hospitals in Ethiopia, located in the Central Gondar zone of Amhara Regional State. It is found 750 km away from Addis Ababa, the capital city of Ethiopia, and is serving more than 7.5 million people living in the Central Gondar Zone and the neighboring zone. It is composed of operating rooms, maternity units, intensive care units (ICUs), a fistula center, 13 different inpatients wards, and outpatient departments. The maternity units of the hospital provide different services such as antenatal, delivery, and postnatal services to women in the reproductive-aged group (15–49 years). According to the hospital directors’ annual reports, around 9,804 neonates were delivered at the University of Gondar Comprehensive Specialized Hospital in the year 2019/20. An institution-based prospective cross-sectional study was conducted from 1 March to 1 May 2020.

All maternal-newborn pairs, who have attended delivery at the University of Gondar Comprehensive Specialized Hospital, were the source population, and those maternal-newborn pairs who have attended delivery during the data collection period were the study population.

All neonates delivered at the University of Gondar Comprehensive Specialized Hospital during the study period were included. Twin newborns were excluded from the study.

The sample size was calculated using a single population proportion formula with the assumption of 11.6% proportion taken from a similar study in Ethiopia (38), considering a 95% confidence level and marginal error of 3%. By adding a 10% non-response rate the required sample size was 481. A systematic random sampling technique was used for the selection of study participants. The client registration book of 2 months before the data collection period was reviewed and then the total number of deliveries during a data collection time was calculated (1,634 delivery in 2 months). To calculate the sampling interval (K), the total estimated number of all births during the study period (1,634) was divided by the sample size (481). The sampling interval (K) was calculated as 1,634/481 = 3. Of the first three study participants, one was chosen randomly. Then, participants in every three intervals were included until the target sample size was achieved.

The dependent variable for this study was low birth weight (defined as a birth weight of newborns < 2,500 g). The independent variables are socio-demographic variables (maternal age, residence, religion, ethnicity, educational status, marital status, occupational status, and monthly income), maternal anthropometric factors (maternal height, weight, and BMI), obstetric- and medical-related factors (gestational age, PIH, parity, birth interval, utilization of ANC visit, birth interval, and chronic medical illness during pregnancy), and newborn factors (sex of the baby and neonatal death). Pregnancy-induced hypertension is defined as the presence of at least one of the following diseases: (A) previous hypertension, (B) gestational hypertension and pre-eclampsia, (C) previous hypertension and superimposed gestational hypertension with proteinuria (39).

To collect the required data, pretested interviewer-administered questionnaires were used. The questionnaire was developed and modified from different literature. It was written in English, converted into Amharic, and translated back into English for suitability and acceptance in approaching respondents. In addition, medical records were reviewed to obtain additional data about maternal and newborn factors. The data were collected by four professional midwives working in the labor wards of the hospital. Anthropometric measurements were done for newborns and mothers using a standard anthropometric technique. The birth weight of a neonate was measured within 1 h of birth using a beam balance scale. The weight of the mothers was measured using a weight measuring scale and recorded accurately to 100 g. Maternal height was measured with calibrated height measuring steel connected to the beam balance and recorded with an accuracy of 0.5 cm. Women were instructed to remain standing up in erect position, with their barefoot close together, while a horizontal headpiece was lowered onto the mother’s heads. Then, maternal BMI was calculated accordingly. The last normal menstrual period was obtained from the mother’s chart since most of the mothers had regular ANC follow-ups. Moreover, if the last normal menstrual period was not documented in the chart, mothers were interviewed during labor. An early pregnancy ultrasound report (≤20 completed weeks of gestation) was also used. Finally, gestational age was determined using either the last normal menstrual period or a chart review of early ultrasound results.

### Data quality control

In total, 3 days of training were provided to data collectors on how to collect the data. Pre-testing of the questionnaire was conducted with 5% of mothers who were delivered in Debark hospital. Daily supervision of the data collectors was performed by the principal investigators. The collected data were checked for completeness and consistency before entry.

### Data processing and analysis

The data were entered into Epidata version 4.6. Then, data were exported and analyzed using STATA version14 software. Descriptive statistics such as percentages, mean, and standard deviations (SD) were used to explain the characteristics of study participants. The findings were presented in the form of tables and text. A chi-square test was used to verify the assumptions. Variables that were passed at the chi-square test were selected for multiple logistic regression analyses. Bivariable and multivariable binary logistic regression analyses were carried out. In the bivariable analysis, variables with a *p*-value of < 0.2 were selected for multivariable binary logistic regression analysis. In the multivariable logistic regression analysis, adjusted odds ratios with corresponding 95% CIs were computed. Finally, variables with a *p-*value of < 0.05 were used to determine factors significantly associated with low birth weight. Model fitness was tested using the Hosmer–Lemeshow test (0.925). The multi-collinearity test between explanatory variables was assessed using variance influencing factor (VIF) and tolerance and the mean VIF was less than 5.

## Results

### Socio-demographic variables of the respondents

A total of 481 respondents were involved in this study with a response rate of 100%. The mean age of the mothers was 28.2 (SD ± 5.4) years. About 85.3% of the study participants were between the age of 21 and 35 years old. Most (96.9%) of the participants were Amhara and 90.8% of them were orthodox religious followers. The majority (93.8%) of respondents were married, and around 47.6% were housewives. Regarding educational status, nearly 35% of the mothers were diploma holders and above. Concerning the income distribution, 10.6% of the mothers had a monthly income of ≤ 1,210 Ethiopian birr (Table 1).

**TABLE 1:** Socio-demographic and anthropometric characteristics of mothers in the University of Gondar Comprehensive Specialized Hospital, Northwest Ethiopia, 2020 (*n* = 481).

Variables	Category	Frequency	Percent (%)
	≤20	29	6
Maternal age (years)	21–35	410	85.3
	>35	42	8.7
	Urban	376	78.2
Residence	Rural	105	21.8
	Amhara	466	96.9
Ethnicity	Tigrie	2	0.4
	Kimant	13	2.7
Marital status	Married	451	93.8
	Single	17	3.5
	Divorced and separated	13	2.7
Religion	Orthodox	437	90.8
	Muslim	44	9.2
	Unable to read and write	88	18.3
Maternal education	read and write only	39	8.1
	Primary Education	67	13.9
	Secondary education	119	24.7
	Diploma and above	168	34.9
	Unable to read and write	71	14.8
Husband education	read and write only	60	12.5
	Primary Education	63	13.1
	Secondary education	93	19.3
	Diploma and above	194	40.3
	Housewife	230	47.6
Maternal occupation	Government employee	123	25.8
	Non-government employee	8	1.7
	Merchant	91	18.9
	Daily laborer	11	2.3
	Students and unemployed	18	3.7
Monthly income in Ethiopian Birr	≤1210	51	10.6
	1211–8970	353	73.4
	>8970	77	16
Number of family members	≤4 members	373	77.5
	>4 members	108	22.5

### Anthropometric characteristics of mothers

In our study, majority (90.3%) of women had a height of >150 cm and nearly three-fourth (78.4%) of mothers had normal BMI (Table 2).

**TABLE 2:** Anthropometric measurement characteristics of the mother in the University of Gondar Comprehensive Specialized Hospital, Northwest Ethiopia, 2020 (*n* = 481).

Variables	Category	Frequency	Percent (%)
Height (cm) Weight (kg)	≤150	42	8.7
	>150	439	91.3
	<50	44	9.2
Maternal BMI(kg/m2)	≥50	437	90.8
	<18.5	15	3.1
	18.5–22.99	377	78.4
	≥25	89	18.5

### Obstetric and medical-related characteristics of mothers

In this study, the majority (95.6%) of the study participants had regular ANC follow-ups during pregnancy and 76.6% of them had at least four visits. Regarding the pregnancy status, the majority (72.6%) of pregnancies were wanted and planned. The majority of (88.2%) mothers had got dietary counseling by health professionals during their ANC follow-up period. Out of 481 mothers, 88.4% were term. One in ten mothers had a chronic medical illness during pregnancy (10%). Concerning the mode of delivery for current pregnancy, 54.1% of women were giving birth *via* spontaneous vaginal delivery (Table 3).

**TABLE 3:** Obstetric and medical-related characteristics of the mother in the University of Gondar Comprehensive Specialized Hospital, Northwest Ethiopia, 2020 (*n* = 481).

Variables	Category	Frequency	Percent (%)
ANC follow up	Yes	460	95.6
	No	21	4.4
Number of ANC visit	<4 visits	107	23.3
	≥4 visits	353	76.7
Pregnancy status	Wanted and planned	349	72.6
	Wanted but unplanned	114	23.7
	Unwanted and unplanned	18	3.7
Dietary counseling during pregnancy	Yes	424	88.2
	No	57	11.8
Parity	Primiparous	181	37.6
	Multiparous	260	54.1
	Grand multiparous	40	8.3
Gestational age at delivery	Preterm	40	8.3
	Term	425	88.4
	Post-term	16	3.3
Birth interval in year	<3	246	51.1
	≥3	235	48.9
Pregnancy-induced hypertension	Yes	54	11.2
	No	427	88.8
Meal frequency per day	<4 times	269	55.9
	≥4 times	212	44.1
Medical illness	Yes	48	10
	No	433	90
Types of medical illness	HIV/AIDS	5	10.4
	Anemia	20	41.7
	Urinary tract infection	8	16.7
	Cardiac disease	3	6.2
	Others*	12	25
Previous history of stillbirth	Yes	42	8.7
	No	439	91.4
Modes of delivery	Spontaneous vaginal delivery	260	54.1
	Cesarean section	205	42.6
	Instrumental delivery	16	3.3

Others* = renal disease and malaria.

### Neonatal characteristics

The mean birth weight of the newborns was 3,027 g (SD ± 584). The average gestational age of the neonates was 39 (SD ± 2) weeks. Out of the total number of poor Apgar score neonates (a score of < 7 at 5 min), 47.1% had low birth weight. Of the total number of birth defect neonates, 33.3% had low birth weight (Table 4).

**TABLE 4:** Neonatal-related variables with the prevalence of low birth weight in the University of Gondar Comprehensive Specialized Hospital, Northwest Ethiopia, 2020 (*n* = 481).

Variables	Category	Low birth weight		Total
		Yes	No	
Sex newborn	Male	35(12.9%)	236(87.1%)	271(100%)
	Female	25(11.9%)	185(88.1%)	210(100%)
Neonatal death	Yes	5(41.7%)	7(58.3%)	12(100%)
	No	55(11.7%)	414(88.3%)	469(100%)
APGAR score	Poor (<7)	16(47.1%)	18(52.9%)	34(100%)
	Good (≥7)	44(9.8%)	403(90.2%)	447(100%)
Congenital malformation	Yes	1(33.3%)	2(66.7%)	3(100%)
	No	60(12.5%)	418(87.5%)	478(100%)

APGAR: Appearance, pulse rate, grimace, activity, respiratory rate.

### Prevalence of low birth weight

The prevalence of low birth weight in this study was 12.5% (95% CI: 9.8, 15.7%). About 45% of the low birth weight neonates were preterm (<37 weeks of gestation).

### Factors associated with low birth weight

Variables including, sex of the newborn, Apgar score, birth interval, congenital malformation, and chronic medical illness were excluded from multivariable analysis because their *p*-value in the bivariable analysis was > 0.2. In the multivariable logistic regression analysis: Maternal age of > 35 years, preterm birth, PIH, maternal BMI of < 18.5 kg/m^2^, and grand multiparity were statistically associated with low birth weight. In this study, mothers who deliver after the age of 35 years had 86% lower odds of low birth weight babies than those mothers between 21 and 35 years old (AOR = 0.14; 95% CI: 0.03,0.7). The odds of being low birth weight in babies born from mothers who gave preterm birth were 38 times greater than low birth weight in babies from those mothers who gave birth ≥ 37 weeks of gestation (AOR = 38; 95% CI: 15.3,93). Mothers with PIH had 2.6 times higher odds of giving low birth weight babies than their counterparts (AOR = 2.6; 95% CI; 1.1, 6.4). The odds of being low birth weight in babies born from mothers whose BMI was less than 18.5 kg/m^2^ were 6.8 times higher than the odds of low weight in babies from mothers with a BMI of > 25 kg/m^2^ (AOR = 6.8; 95% CI: 1.5, 31.1). The odds of being low birth weight in neonates born from grand multiparous women were four times higher than the odds of low birth weight in neonates from multiparous women (AOR = 4.2; 95% CI:1.2,16) (Table 5).

**TABLE 5:** Factors associated with low birth weight among newborns delivered at the University of Gondar Comprehensive Specialized Hospital, Northwest Ethiopia, 2020 (*n* = 481).

		Low birth weight		COR	AOR
Variables	Category	Yes (%)	No (%)	(95% CI)	(95% CI)
Maternal age	≤20	7 (11.7%)	22 (5.2%)	2.3 (0.9,5)	2.8(0.8, 9.7)
(years)	21–35	50 (83.3%)	360 (85.5%)	1	1
	>35	3 (5%)	39 (9.3)%	0.6 (0.1, 0.9)	0.14(0.03, 0.7)*
Residence	Urban	41 (68.3%)	335(79.6%)	1	1
	Rural	19 (36.7%)	86(20.4%)	1.8 (0.9, 3)	0.7(0.2, 1.8)
	Unable to read and write	20(33.3%)	68(16.2%)	3.2(1.5, 6.7)	2.5(0.7, 8.5)
Maternal	read and write only	5 (8.3%)	34 (8%)	1.6 (0.5,4.8)	0.8(0.2,3.4)
education	Primary Education	8 (13.4%)	59(14%)	1.4 (0.6,3.8)	0.8(0.3,2.6)
	Secondary education	13 (21.7%)	106(25.2%)	1.3 (0.6,3.9)	1(0.4,2.9)
	Diploma and above	14 (23.3%)	154(36.6%)	1	1
Monthly income in Ethiopian Birr	≤1210	10 (16.7%)	41(9.7%)	4.4(1.3,15)	1.9(0.3,10)
	1211–8970	46 (76.6%)	307(72.9%)	2.7(0.9,7.8)	1.5(0.4,5.6)
	>8970	4 (6.7%)	73(17.4%)	1	1
ANC visit	Yes	55 (9.7%)	405(96.2%)	1	1
	No	5 (8.3%)	16(2.8%)	2.3(0.8,6.5)	1.5(0.3,6.7)
Pregnancy status	Wanted and planned	38 (63.3%)	311(73.9%)	1	1
	Wanted but unplanned	19 (31.7%)	95(22.6%)	1.6(0.9,2.9)	0.9(0.4,2.2)
	Unwanted and unplanned	3 (5%)	15(3.5%)	1.6(0.5,5.9)	1 (0.2,4.9)
	Primiparous	27(45%)	154(36.6%)	1.6(0.9,2.9)	1.9(0.8,4.4)
Parity	Multiparous	25(41.7%)	235(55.8%)	1	1
	Grand multiparous	8(13.3%)	32 (7.6%)*	2.4(1.01,5.9)	4.2(1.2,16)*
Gestational age (weeks)	<37	27(45%)	13(3.1%)	25 (12,54)	38(15.3,93.0)**
	≥37	33(55%)	408(96.9%)	1	1
PIH	Yes	12(20%)	42(10%)	2.3(1.2,4.6)	2.6(1.1,6.4)*
	No	48(80%)	379(90%)	1	1
Meal frequency per day	<4 times	42(70%)	227(53.9%)	2(1.1,3.5)	1.4(0.6,3.1)
	≥4 times	18(30%)	194(46.1%)	1	1
Maternal BMI (kg/m2)	<18.5	6(10%)	9(2.1%)	5.9(1.7,20)	6.8(1.5,31.1)*
	18.5–22.99	45(75%)	332(78.9%)	1.2(0.6,2.5)	0.9(0.3,2.5)
	≥25	9(15%)	80(19%)	1	1
Neonatal death	Yes	5(8.3%)	7(1.7%)	5.4(1.7,17.5)*	2.4 (0.4,14)
	No	55(91.7%)	414(98.3%)	1	1

*Statistically significant at p < 0.05, ** p < 0.001.

The Hosmer–Lemeshow test of goodness-of-fit = 0.925.

1, reference category.

## Discussion

In this study, the overall prevalence of low birth weight was 12.5% [95% CI: 9.8, 15.7%] which is in agreement with the study conducted in Dangla (14) and the worldwide prevalence of low birth weight (24). The prevalence of low birth weight in this study is lower than the reports in Wolaita Sodo, Ethiopia (1), Kersa, Ethiopia (15), and India (9). The variation in the prevalence of low birth weight among different studies might be explained because of variation in methodology, study setup, study time, and study design. The difference between the present study and the Wolaita Sodo report might be due to the variation in methodology, in the way that, the current study considered only singleton deliveries which decrease the burden of low birth weight. The difference in the study set-up may have been a reason for variations since the present study was carried out in an urban area, whereas the study done in Kersa was in a rural area. Besides, the possible explanation for this variation is also the time difference between the studies. There is a 10-year time gap between the present study and the study done in Kersa, Ethiopia. Thus, increasing healthcare coverage and quality of care over time may result in a reduction in low birth weight. The other reason for variation may be the study design. For example, the Indian study was a community-based study, but the present study is a hospital-based one, particularly, in healthcare facilities. Women who were delivered in healthcare facilities are assumed to obtain regular ANC follow-up and good interventions, which drastically decrease low birth weight (29, 40).

In the current study, the prevalence of low birth weight infants was higher than in the studies conducted in Jimma (20), Ghana (2), Axum (25), and Yazd, Iran (41). The possible explanation for this variation might be due to a difference in the nutritional status of women and the healthcare provider’s commitment to ANC service provision, particularly on the nutritional counseling during pregnancy (1). In addition, the variation between the present study and a study done in Iran might be due to differences in socio-demographic factors, health service delivery system, and the approaches to managing women during the ANC follow-up period.

In the multivariable binary logistic regression analysis parity, maternal age, gestational age, pregnancy-induced hypertension, and maternal BMI were significantly associated with low birth weight. Mothers who deliver after the age of 35 years had lower odds to have a low birth weight baby which is similar to other studies (2, 11, 37). This might be because higher age groups might be less likely to deliver low birth weight infants usually as the pregnancy might be planned and wanted, which leads to giving more attention to the dietary value and healthcare services utilization and increased awareness of pregnancy’s danger signs and main risk factors (37). In this study, grand multiparous women had higher odds to deliver low birth weight infants. This study is supported by the finding conducted in Jimma (20), Wolaita Sodo (1), East Gojam (27), and Ghana (11). This can be justified by grand multiparty mothers are at high risk of several adverse fetal and maternal outcomes such as abnormal implantation, abruption placenta, instrumental delivery, postpartum hemorrhage, preterm birth, and neonatal and maternal admission to intensive care unit, some of which may have an effect on fetal development including their birth weight (42). Besides, the odds of women who gave birth before 37 weeks of gestation had greater odds to deliver a low birth weight infant. The finding was in line with the studies carried out in Dangla (14), Jimma (20), Addis Ababa (31), and Tigray region (25). The possible explanation for this is as the gestational age of the fetus falls under the acceptable range of time, the birth weight of the fetus decreased significantly because of the premature birth (25). Interestingly, women having pregnancy-induced hypertension had higher odds of low birth weight delivery, which is consistent with the studies done in Wolaita Sodo (1), East Gojam (27), and Addis Ababa (31). This might be due to a reduction in uteroplacental blood perfusion as a result of vasoconstriction of blood vessels during a hypertensive state, which leads to intrauterine growth restriction and premature birth which becomes low birth weight (27, 43). Regarding the maternal BMI, those women who had a BMI of < 18.5 kg/m2 were more likely to give low birth weight infants. This is in agreement with studies conducted elsewhere (32, 37, 44). This might be because maternal BMI is one of the main parameters to assess nutritional status. Those mothers who had a BMI of less than 18.5 kg/m^2^ indicate the presence of under-nutrition that shows chronic malnutrition in adults. This might result in the impairment of fetal growth and development in the uterus. Therefore, low birth weight might easily occur. Malnutrition in pregnancy has also been related to decrease placental weight and surface area, which might restrict the transport of nutrients from the placenta to the fetus. Furthermore, poor maternal nutrition leads to a decrement in serum levels of hormones, such as leptin and estrogen, that results in fetal growth restriction (37).

As the study design was cross-sectional, we did not determine the reverse causality between the dependent and different independent variables, and this is one of the main limitations of this cross-sectional study. The study did not assess some factors such as lifestyle factors (dietary habit, level of physical activity, etc.), intrauterine growth retardation, small for gestational age, and weight gain during pregnancy.

## Conclusion

The prevalence of low birth weight in this study was higher than in previous studies, implying that it continues to be a major public health concern. This study showed that preterm birth, PIH, grand multiparity, and BMI of < 18.5 kg/m^2^ were the major associated factors of low birth weight. However, maternal age > 35 years was a protective factor. Therefore, early identification and management of women with PIH, tackling prematurity, and preventing maternal malnutrition through nutrition education during the ANC follow-up period are recommended to reduce the burden of low birth weight.

## Data availability statement

The raw data supporting the conclusions of this article will be made available by the authors, without undue reservation.

## Ethics statement

The studies involving human participants were reviewed and approved by the Ethical review board University of Gondar. Written informed consent to participate in this study was provided by the participants’ legal guardian/next of kin.

## Author contributions

DA and MW: conceptualization, formal analysis, methodology, resources, software, supervision, validation, visualization, and writing—original draft. DA: data curator and investigation. Both authors read and approved the final manuscript.
